# *catena*-Poly[[μ_2_-aqua-diaqua­bis­(μ_4_-pyridazine-3,6-dicarboxyl­ato)tetra­lithium] monohydrate]

**DOI:** 10.1107/S1600536811038992

**Published:** 2011-09-30

**Authors:** Wojciech Starosta, Janusz Leciejewicz

**Affiliations:** a Institute of Nuclear Chemistry and Technology, ul.Dorodna 16, 03-195 Warszawa, Poland

## Abstract

In the polymeric structure of the title compound {[Li_2_(C_6_H_2_N_2_O_4_)_2_Li(H_2_O)_2_Li(H_2_O)]·H_2_O}_*n*_, the coordination of two independent Li^I^ ions is distorted trigonal–bipyramidal and that of the other two independent Li^I^ ions is distorted tetra­hedral. The former two Li^I^ ions are bridged by hetero-ring N atoms of two independent pyridazine-3,6-dicarboxyl­ate ligands, making a dimeric moiety. The carboxyl­ato-O atoms of both bidentate ligands bridge the dimers to adjacent independent aqua-coordinated Li^I^ ions, forming mol­ecular ribbons. The latter are bridged by ligand carboxyl­ato and aqua O atoms, forming mol­ecular layers parallel to (100) which are held together by an extended system of O—H⋯O hydrogen bonds.

## Related literature

For the crystal structure of a Li^I^ complex with water and pyridazine-3,6-dicarboxyl­ate ligands, see: Starosta & Leciejewicz (2010 ▶). 
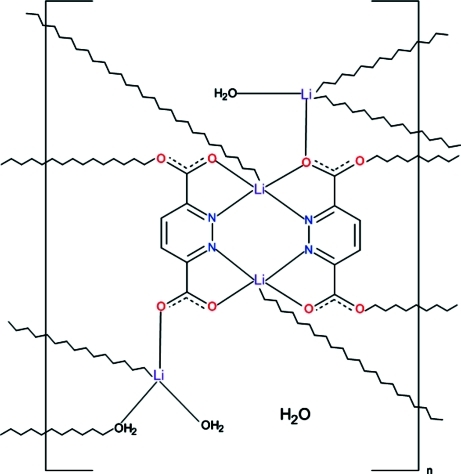


## Experimental

### 

#### Crystal data

[Li_4_(C_6_H_2_N_2_O_4_)_2_(H_2_O)_3_]·H_2_O
*M* *_r_* = 432.02
Triclinic, 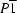

*a* = 7.1460 (14) Å
*b* = 10.553 (2) Å
*c* = 11.849 (2) Å
α = 74.76 (3)°
β = 88.84 (3)°
γ = 82.66 (3)°
*V* = 855.0 (3) Å^3^
*Z* = 2
Mo *K*α radiation
μ = 0.15 mm^−1^
*T* = 293 K
0.63 × 0.11 × 0.10 mm

#### Data collection

Kuma KM4 four-circle diffractometer
Absorption correction: analytical (*CrysAlis RED*; Oxford Diffraction, 2008 ▶) *T*_min_ = 0.984, *T*_max_ = 0.987
5238 measured reflections
4991 independent reflections
3628 reflections with *I* > 2σ(*I*)
*R*_int_ = 0.040
3 standard reflections every 200 reflections  intensity decay: 2.3%

#### Refinement

*R*[*F* ^2^ > 2σ(*F* ^2^)] = 0.045
*wR*(*F* ^2^) = 0.142
*S* = 1.07
4991 reflections
321 parameters
H atoms treated by a mixture of independent and constrained refinement
Δρ_max_ = 0.52 e Å^−3^
Δρ_min_ = −0.40 e Å^−3^

### 

Data collection: *KM-4 Software* (Kuma, 1996 ▶); cell refinement: *KM-4 Software*; data reduction: *DATAPROC* (Kuma, 2001 ▶); program(s) used to solve structure: *SHELXS97* (Sheldrick, 2008 ▶); program(s) used to refine structure: *SHELXL97* (Sheldrick, 2008 ▶); molecular graphics: *SHELXTL* (Sheldrick, 2008 ▶); software used to prepare material for publication: *SHELXTL*.

## Supplementary Material

Crystal structure: contains datablock(s) I, New_Global_Publ_Block. DOI: 10.1107/S1600536811038992/vm2123sup1.cif

Structure factors: contains datablock(s) I. DOI: 10.1107/S1600536811038992/vm2123Isup2.hkl

Additional supplementary materials:  crystallographic information; 3D view; checkCIF report

## Figures and Tables

**Table 1:** Hydrogen-bond geometry (Å, °)

*D*—H⋯*A*	*D*—H	H⋯*A*	*D*⋯*A*	*D*—H⋯*A*
O31—H312⋯O14^i^	0.75 (3)	2.11 (3)	2.8572 (17)	171 (3)
O42—H421⋯O1	0.82 (3)	1.97 (3)	2.768 (2)	165 (3)
O42—H422⋯O23^ii^	0.86 (3)	1.95 (3)	2.7676 (16)	158 (2)
O1—H11⋯O24^iii^	0.91 (3)	2.00 (3)	2.9036 (19)	175 (2)
O31—H311⋯O11	0.81 (3)	1.92 (3)	2.7117 (17)	163 (2)
O41—H442⋯O14^iv^	0.84 (2)	2.10 (2)	2.9306 (18)	167 (2)
O41—H441⋯O12^v^	0.84 (3)	1.92 (3)	2.7607 (16)	172 (2)

Symmetry codes: (i) 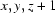; (ii) 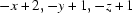; (iii) 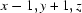; (iv) 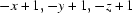; (v) 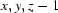.

**Fig. 1.**
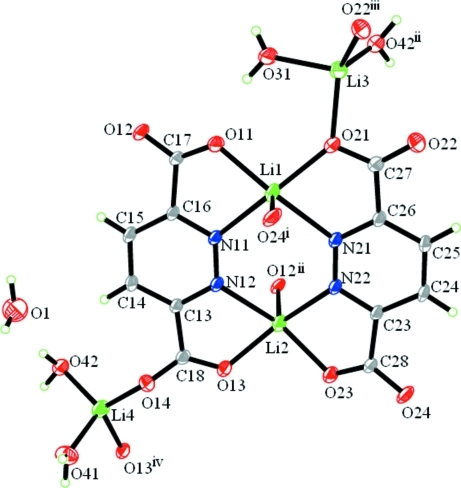
A structural unit of the title compound with atom labelling scheme and 50% probability displacement ellipsoids. Symmetry codes: i -x + 2, -y, -z + 2; ii -x + 2, -y + 1, -z + 2; iii -x + 2, -y, -z + 3; iv -x + 2, -y + 1, -z + 1.

**Fig. 2.**
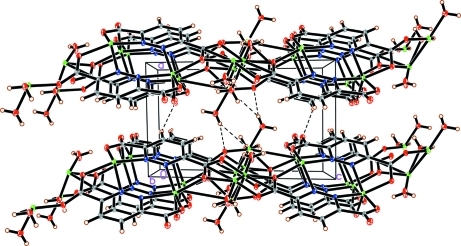
Packing diagram of the structure viewed along the b axis.

## supplementary crystallographic information

### Comment

Li^I^ ion forms with pyridazine-3,6-dicarboxylate and water ligands a complex composed of centrosymmetric monomers in which the metal ion is chelated by two singly deprotonated ligand molecules and two water O atoms giving rise to octahedral coordination with aqua O atoms at the axial positions. A proton located between adjacent aqua O atoms, apart from maintaining charge balance, bridges the monomers *via* strong centrosymmetric hydrogen bonds to form catenated ribbons (Starosta & Leciejewicz, 2010). It has been of interest to study structural changes brought about by removal of the bridging protons. Hydrazine has been selected as the deprotonating agent. The structure of a complex obtained when the amount of added hydrazine was very small is described in this report.

The title compound is a polymeric complex with four symmetry independent Li ions in the asymmetric unit. Two of them show distorted trigonal bipyramidal geometry, the other two exhibit distorted tetrahedral coordination environment. The asymmetric unit contains also two pyridazine-3,6-dicarboxylate ligand molecules (PY1 with atoms labels starting with 1 and PY2 with atoms labels starting with 2), three coordinated water molecules and a solvation water molecule (Fig.1). The equatorial plane of the Li1 coordination polyhedron is composed of atoms O11, N21, O24^i^. The Li1 ion is 0.0285 (2) Å out of the plane, atoms O21 and N11 are at axial positions. Li2 ion is shifted by 0.0186 (2) Å from the basal plane composed of atoms O12^ii^, O23, N12; atoms O13 and N22 make the apices. Li3 ion is coordinated by atoms O21, O22^iii^, O31, O42^ii^ at the apices of a distorted tetrahedron while the coordination tetrahedron of the Li4 ion is composed of atoms O13^iv^, O14, O41, O42 [Symmetry codes: ^i^ -*x* + 2, -*y*, -*z* + 2; ^ii^ -*x* + 2, -*y* + 1, -*z* + 2; ^iii^ -*x* + 2, -*y*, -*z* + 3; ^iv^ -*x* + 2, -*y* + 1, -*z* + 1]. The Li—O and Li—N bond distances are close to those observed in the other Li complex with the title ligand (Starosta & Leciejewicz, 2010). Both pyridazine rings are planar with r.m.s. deviation of 0.0154 (2)Å and 0.0123 (2)Å for the ring PY1 and PY2, respectively. Carboxylate groups C17/O11/O12 and C18/O13/O14 make with the hetero-ring PY1 dihedral angles of 14.3 (1)° and 22.2 (2)°, respectively. Dihedral angles formed with the hetero-ring PY2 by carboxylate groups C27/O21/O22 and C28/O23/O24 amount to 3.8 (1)° and 17.2 (2)°, respectively. The Li1 and Li2 ions bridged by hetero-ring N atoms donated by both ligands along the Li1—N11—N12—Li2—N22—N21—Li1 pathway form a dimeric moiety. The C27/O21/O22 and C27^iii^/O21^iii^/O22^iii^ groups act as bidentate bridge between the Li3 and Li3^iii^ ions to form a loop which joins two dimers *via* O21 and O21^iii^ atoms since the latter are also bonded to the Li1 and Li1^iii^ ions, respectively. A similar loop bridges the dimers from the other side as the bidentate O13 atom links the Li2 and Li4^iv^ ions. A molecular ribbon propagating along the *c* direction can be visualized (Fig. 2). The ribbons bridged by carboxylate and coordinated water O atoms form molecular layers which are parallel to the *bc* plane and stacked along the *a* axis direction. The bridging of ribbons proceeds *via* carboxylato O12 and O24 atoms: atom O12 is coordinated to the Li2^ii^ atom in an adjacent ribbon, while the Li2 ion by the O12^ii^ atom from the same adjacent ribbon. The O24 atom is chelated to the Li1^i^ ion in the other adjacent ribbon, while the O24^i^ atom is coordinated to the Li1 ion. In addition, pairs of ribbons are bridged by coordinated aqua O42 atoms *via* Li4—O42—Li3^ii^ and Li3—O42^ii^—Li4^ii^ links. An extended system of hydrogen bonds in which coordinated water molecules are donors and carboxylato O atoms in adjacent layers act as acceptors, maintains the stability of the structure (Table 1). Two intra-molecular hydrogen bonds are also observed.

### Experimental

The title complex was obtained by adding three drops of hydrazine to the aqueous solution containing *ca* 1 mmol of the complex previously synthetized (Starosta & Leciejewicz, 2010). The solution was kept at 320 K with constant stirring for 6 h, then left to evaporate at room temperature. Colorless single-crystal columns were washed with cold ethanol and dried in the air.

### Refinement

Water hydrogen atoms were located in a difference map and refined isotropically while H atoms attached to pyridazine-ring C atoms were positioned at calculated positions and were treated as riding on the parent atoms, with C—H=0.93 Å and *U*_iso_(H)=1.2*U*_eq_(C).

### Crystal data

**Table:**

[Li_4_(C_6_H_2_N_2_O_4_)_2_(H_2_O)_3_]·H_2_O	*Z* = 2
*M**_r_* = 432.02	*F*(000) = 440
Triclinic, *P*1	*D*_x_ = 1.678 Mg m^−^^3^
Hall symbol: -P 1	Mo *K*α radiation, λ = 0.71073 Å
*a* = 7.1460 (14) Å	Cell parameters from 25 reflections
*b* = 10.553 (2) Å	θ = 6–15°
*c* = 11.849 (2) Å	µ = 0.15 mm^−^^1^
α = 74.76 (3)°	*T* = 293 K
β = 88.84 (3)°	Columns, colourless
γ = 82.66 (3)°	0.63 × 0.11 × 0.10 mm
*V* = 855.0 (3) Å^3^	

### Data collection

**Table:**

Kuma KM4 four-circle diffractometer	3628 reflections with *I* > 2σ(*I*)
Radiation source: fine-focus sealed tube	*R*_int_ = 0.040
graphite	θ_max_ = 30.1°, θ_min_ = 1.8°
profile data from ω/2θ scans	*h* = −10→9
Absorption correction: analytical (*CrysAlis RED*; Oxford Diffraction, 2008)	*k* = −14→0
*T*_min_ = 0.984, *T*_max_ = 0.987	*l* = −16→16
5238 measured reflections	3 standard reflections every 200 reflections
4991 independent reflections	intensity decay: 2.3%

### Refinement

**Table:**

Refinement on *F*^2^	Primary atom site location: structure-invariant direct methods
Least-squares matrix: full	Secondary atom site location: difference Fourier map
*R*[*F*^2^ > 2σ(*F*^2^)] = 0.045	Hydrogen site location: inferred from neighbouring sites
*wR*(*F*^2^) = 0.142	H atoms treated by a mixture of independent and constrained refinement
*S* = 1.07	*w* = 1/[σ^2^(*F*_o_^2^) + (0.0933*P*)^2^ + 0.1387*P*] where *P* = (*F*_o_^2^ + 2*F*_c_^2^)/3
4991 reflections	(Δ/σ)_max_ = 0.001
321 parameters	Δρ_max_ = 0.52 e Å^−^^3^
0 restraints	Δρ_min_ = −0.40 e Å^−^^3^

### Special details

**Table:**

Geometry. All e.s.d.'s (except the e.s.d. in the dihedral angle between two l.s. planes) are estimated using the full covariance matrix. The cell e.s.d.'s are taken into account individually in the estimation of e.s.d.'s in distances, angles and torsion angles; correlations between e.s.d.'s in cell parameters are only used when they are defined by crystal symmetry. An approximate (isotropic) treatment of cell e.s.d.'s is used for estimating e.s.d.'s involving l.s. planes.
Refinement. Refinement of *F*^2^ against ALL reflections. The weighted *R*-factor *wR* and goodness of fit *S* are based on *F*^2^, conventional *R*-factors *R* are based on *F*, with *F* set to zero for negative *F*^2^. The threshold expression of *F*^2^ > σ(*F*^2^) is used only for calculating *R*-factors(gt) *etc*. and is not relevant to the choice of reflections for refinement. *R*-factors based on *F*^2^ are statistically about twice as large as those based on *F*, and *R*- factors based on ALL data will be even larger.

### Fractional atomic coordinates and isotropic or equivalent isotropic displacement parameters (Å^2^)

**Table:**

	*x*	*y*	*z*	*U*_iso_*/*U*_eq_	
O12	0.66029 (14)	0.62545 (9)	1.13840 (8)	0.0237 (2)	
N12	0.89610 (15)	0.37798 (10)	0.88294 (9)	0.0187 (2)	
O13	1.03460 (14)	0.36050 (9)	0.67631 (8)	0.0239 (2)	
O42	0.76213 (16)	0.82985 (10)	0.46053 (10)	0.0275 (2)	
O11	0.76083 (16)	0.40826 (9)	1.20181 (8)	0.0280 (2)	
N21	1.11362 (15)	0.11238 (10)	1.10459 (9)	0.0192 (2)	
O23	1.25615 (16)	0.09387 (9)	0.78141 (8)	0.0264 (2)	
O14	0.79238 (14)	0.50339 (10)	0.58467 (8)	0.0261 (2)	
O24	1.33640 (15)	−0.12661 (9)	0.84009 (9)	0.0272 (2)	
N11	0.85009 (15)	0.39281 (10)	0.98916 (9)	0.0183 (2)	
N22	1.16009 (15)	0.10114 (10)	0.99701 (9)	0.0184 (2)	
C26	1.17284 (17)	0.01344 (11)	1.19732 (10)	0.0179 (2)	
C17	0.71991 (17)	0.51348 (11)	1.12415 (10)	0.0176 (2)	
C16	0.74862 (16)	0.50523 (11)	0.99854 (10)	0.0162 (2)	
C15	0.67929 (18)	0.60722 (12)	0.90249 (11)	0.0216 (2)	
H15	0.6104	0.6847	0.9118	0.026*	
C28	1.28781 (17)	−0.01463 (12)	0.85721 (11)	0.0183 (2)	
C23	1.25859 (16)	−0.01031 (11)	0.98389 (10)	0.0168 (2)	
C18	0.89136 (17)	0.44348 (11)	0.67313 (10)	0.0182 (2)	
C13	0.83030 (17)	0.47216 (11)	0.78829 (10)	0.0172 (2)	
C14	0.71722 (19)	0.58842 (12)	0.79303 (11)	0.0221 (3)	
H14	0.6695	0.6505	0.7255	0.027*	
Li3	0.9854 (4)	0.1617 (2)	1.4735 (2)	0.0260 (5)	
Li2	1.1410 (4)	0.2530 (2)	0.8362 (2)	0.0252 (5)	
Li1	0.8796 (4)	0.2444 (2)	1.1597 (2)	0.0264 (5)	
O41	0.55920 (17)	0.61557 (13)	0.36637 (10)	0.0346 (3)	
O1	0.4656 (2)	0.86090 (18)	0.60889 (14)	0.0508 (4)	
Li4	0.7754 (4)	0.6481 (2)	0.4458 (2)	0.0276 (5)	
H441	0.580 (3)	0.615 (2)	0.296 (2)	0.051 (7)*	
H442	0.468 (3)	0.573 (2)	0.391 (2)	0.051 (7)*	
C24	1.32555 (18)	−0.11597 (11)	1.07838 (11)	0.0211 (2)	
H24	1.3954	−0.1921	1.0670	0.025*	
C25	1.28368 (19)	−0.10253 (12)	1.18890 (11)	0.0217 (2)	
H25	1.3275	−0.1681	1.2553	0.026*	
C27	1.10638 (18)	0.03368 (12)	1.31489 (11)	0.0206 (2)	
O21	1.00788 (16)	0.14240 (10)	1.31261 (9)	0.0292 (2)	
O22	1.15259 (16)	−0.05770 (10)	1.40266 (9)	0.0310 (2)	
O31	0.8353 (3)	0.32916 (12)	1.43507 (11)	0.0528 (4)	
H311	0.799 (3)	0.366 (2)	1.368 (2)	0.053 (7)*	
H11	0.425 (4)	0.870 (3)	0.680 (2)	0.058 (7)*	
H422	0.750 (3)	0.874 (3)	0.389 (2)	0.052 (7)*	
H421	0.664 (4)	0.847 (3)	0.494 (3)	0.076 (9)*	
H312	0.812 (4)	0.374 (3)	1.474 (2)	0.068 (8)*	
H12	0.391 (6)	0.835 (4)	0.577 (4)	0.135 (17)*	

### Atomic displacement parameters (Å^2^)

**Table:**

	*U*^11^	*U*^22^	*U*^33^	*U*^12^	*U*^13^	*U*^23^
O12	0.0342 (5)	0.0132 (4)	0.0244 (4)	0.0040 (3)	0.0013 (4)	−0.0094 (3)
N12	0.0258 (5)	0.0115 (4)	0.0171 (5)	0.0034 (4)	0.0014 (4)	−0.0036 (4)
O13	0.0291 (5)	0.0205 (4)	0.0199 (4)	0.0073 (4)	0.0015 (3)	−0.0063 (3)
O42	0.0341 (5)	0.0226 (5)	0.0253 (5)	0.0030 (4)	−0.0001 (4)	−0.0086 (4)
O11	0.0496 (6)	0.0141 (4)	0.0175 (4)	0.0058 (4)	−0.0002 (4)	−0.0037 (3)
N21	0.0270 (5)	0.0116 (4)	0.0172 (5)	0.0029 (4)	0.0023 (4)	−0.0035 (4)
O23	0.0423 (6)	0.0151 (4)	0.0196 (4)	0.0039 (4)	0.0012 (4)	−0.0040 (3)
O14	0.0323 (5)	0.0243 (5)	0.0183 (4)	0.0049 (4)	−0.0022 (4)	−0.0036 (3)
O24	0.0394 (6)	0.0148 (4)	0.0290 (5)	0.0023 (4)	0.0047 (4)	−0.0116 (4)
N11	0.0259 (5)	0.0113 (4)	0.0167 (5)	0.0027 (4)	0.0015 (4)	−0.0044 (3)
N22	0.0257 (5)	0.0107 (4)	0.0172 (4)	0.0029 (4)	0.0021 (4)	−0.0034 (3)
C26	0.0236 (5)	0.0107 (5)	0.0185 (5)	0.0007 (4)	0.0017 (4)	−0.0036 (4)
C17	0.0228 (5)	0.0128 (5)	0.0180 (5)	0.0010 (4)	−0.0001 (4)	−0.0067 (4)
C16	0.0211 (5)	0.0095 (5)	0.0179 (5)	0.0011 (4)	0.0006 (4)	−0.0048 (4)
C15	0.0279 (6)	0.0128 (5)	0.0221 (6)	0.0057 (4)	0.0005 (5)	−0.0053 (4)
C28	0.0218 (5)	0.0137 (5)	0.0202 (5)	0.0008 (4)	0.0012 (4)	−0.0074 (4)
C23	0.0206 (5)	0.0106 (5)	0.0188 (5)	0.0002 (4)	0.0010 (4)	−0.0045 (4)
C18	0.0238 (5)	0.0130 (5)	0.0173 (5)	−0.0006 (4)	0.0020 (4)	−0.0039 (4)
C13	0.0210 (5)	0.0129 (5)	0.0167 (5)	0.0013 (4)	0.0005 (4)	−0.0041 (4)
C14	0.0301 (6)	0.0139 (5)	0.0185 (5)	0.0069 (4)	−0.0016 (4)	−0.0018 (4)
Li3	0.0390 (13)	0.0175 (10)	0.0202 (10)	−0.0006 (9)	0.0014 (9)	−0.0039 (8)
Li2	0.0346 (12)	0.0147 (10)	0.0235 (10)	0.0050 (9)	0.0004 (9)	−0.0043 (8)
Li1	0.0370 (12)	0.0139 (10)	0.0266 (11)	0.0059 (9)	−0.0005 (9)	−0.0066 (8)
O41	0.0340 (6)	0.0450 (7)	0.0264 (5)	−0.0075 (5)	0.0030 (4)	−0.0113 (5)
O1	0.0392 (7)	0.0700 (10)	0.0429 (8)	−0.0024 (7)	0.0095 (6)	−0.0170 (7)
Li4	0.0376 (13)	0.0201 (11)	0.0239 (11)	−0.0003 (9)	0.0035 (9)	−0.0052 (9)
C24	0.0267 (6)	0.0108 (5)	0.0237 (6)	0.0055 (4)	0.0005 (5)	−0.0047 (4)
C25	0.0295 (6)	0.0119 (5)	0.0202 (5)	0.0047 (4)	−0.0006 (4)	−0.0013 (4)
C27	0.0262 (6)	0.0169 (5)	0.0186 (5)	−0.0005 (4)	0.0014 (4)	−0.0058 (4)
O21	0.0442 (6)	0.0206 (5)	0.0209 (4)	0.0102 (4)	−0.0001 (4)	−0.0091 (4)
O22	0.0451 (6)	0.0225 (5)	0.0198 (4)	0.0022 (4)	0.0040 (4)	0.0010 (4)
O31	0.1059 (13)	0.0231 (5)	0.0228 (5)	0.0250 (7)	−0.0087 (6)	−0.0090 (5)

### Geometric parameters (Å, °)

**Table:**

Li1—O21	2.018 (3)	N11—C16	1.3386 (14)
Li1—O24^i^	2.101 (3)	N22—C23	1.3352 (15)
N11—Li1	2.202 (3)	C26—C25	1.3959 (16)
O11—Li1	2.005 (2)	C26—C27	1.5217 (17)
N21—Li1	2.237 (3)	C17—C16	1.5219 (16)
Li2—O12^ii^	2.107 (3)	C16—C15	1.3936 (17)
O13—Li2	2.040 (3)	C15—C14	1.3792 (17)
N12—Li2	2.206 (3)	C15—H15	0.9300
N22—Li2	2.137 (3)	C28—C23	1.5231 (16)
O23—Li2	2.029 (2)	C23—C24	1.3982 (17)
Li3—O31	1.896 (3)	C18—C13	1.5176 (16)
Li3—O22^iii^	1.924 (3)	C13—C14	1.3937 (16)
Li3—O21	1.971 (3)	C14—H14	0.9300
Li3—O42^ii^	2.002 (3)	Li3—Li4^ii^	3.133 (4)
O42—Li3^ii^	2.002 (3)	Li3—Li3^iii^	3.283 (5)
Li2—Li4^iv^	3.295 (4)	O41—Li4	1.938 (3)
O12—C17	1.2569 (14)	O41—H441	0.84 (3)
O12—Li2^ii^	2.107 (3)	O41—H442	0.84 (2)
N12—C13	1.3352 (16)	O1—H11	0.91 (3)
N12—N11	1.3380 (14)	O1—H12	0.77 (4)
O13—C18	1.2535 (15)	Li4—O13^iv^	1.976 (3)
O13—Li4^iv^	1.976 (3)	Li4—Li3^ii^	3.133 (4)
O42—Li4	1.961 (3)	Li4—Li2^iv^	3.295 (4)
O42—H422	0.86 (3)	Li4—H422	2.28 (3)
O42—H421	0.82 (3)	C24—C25	1.3768 (18)
O11—C17	1.2474 (15)	C24—H24	0.9300
N21—C26	1.3339 (16)	C25—H25	0.9300
N21—N22	1.3423 (14)	C27—O22	1.2353 (16)
O23—C28	1.2531 (16)	C27—O21	1.2612 (16)
O14—C18	1.2515 (16)	O22—Li3^iii^	1.924 (3)
O14—Li4	1.922 (3)	O31—H311	0.81 (3)
O24—C28	1.2556 (14)	O31—H312	0.75 (3)
O24—Li1^i^	2.101 (3)		
			
C17—O12—Li2^ii^	118.39 (11)	Li4^ii^—Li3—Li3^iii^	133.53 (13)
C13—N12—N11	119.30 (10)	O23—Li2—O13	95.51 (11)
C13—N12—Li2	109.89 (10)	O23—Li2—O12^ii^	113.67 (12)
N11—N12—Li2	127.57 (10)	O13—Li2—O12^ii^	99.66 (11)
C18—O13—Li4^iv^	128.88 (11)	O23—Li2—N22	78.96 (9)
C18—O13—Li2	118.04 (11)	O13—Li2—N22	157.39 (15)
Li4^iv^—O13—Li2	110.25 (11)	O12^ii^—Li2—N22	102.64 (11)
Li4—O42—Li3^ii^	104.46 (12)	O23—Li2—N12	151.64 (14)
Li4—O42—H422	100.8 (17)	O13—Li2—N12	77.64 (9)
Li3^ii^—O42—H422	111.7 (16)	O12^ii^—Li2—N12	94.65 (10)
Li4—O42—H421	109 (2)	N22—Li2—N12	96.76 (11)
Li3^ii^—O42—H421	121 (2)	O23—Li2—Li4^iv^	71.49 (9)
H422—O42—H421	108 (2)	O13—Li2—Li4^iv^	34.23 (7)
C17—O11—Li1	120.35 (11)	O12^ii^—Li2—Li4^iv^	87.26 (10)
C26—N21—N22	119.26 (10)	N22—Li2—Li4^iv^	150.33 (11)
C26—N21—Li1	108.68 (10)	N12—Li2—Li4^iv^	110.40 (10)
N22—N21—Li1	129.45 (10)	O11—Li1—O21	100.76 (12)
C28—O23—Li2	117.34 (10)	O11—Li1—O24^i^	106.87 (12)
C18—O14—Li4	144.59 (13)	O21—Li1—O24^i^	98.88 (12)
C28—O24—Li1^i^	116.63 (11)	O11—Li1—N11	77.16 (9)
N12—N11—C16	119.33 (10)	O21—Li1—N11	155.87 (15)
N12—N11—Li1	129.04 (10)	O24^i^—Li1—N11	104.75 (12)
C16—N11—Li1	110.95 (10)	O11—Li1—N21	155.62 (15)
C23—N22—N21	119.79 (10)	O21—Li1—N21	76.83 (9)
C23—N22—Li2	111.01 (10)	O24^i^—Li1—N21	97.44 (10)
N21—N22—Li2	128.28 (10)	N11—Li1—N21	95.10 (11)
N21—C26—C25	123.30 (11)	Li4—O41—H441	113.3 (16)
N21—C26—C27	115.14 (10)	Li4—O41—H442	132.4 (15)
C25—C26—C27	121.54 (11)	H441—O41—H442	109 (2)
O11—C17—O12	127.16 (11)	H11—O1—H12	112 (3)
O11—C17—C16	116.17 (10)	O14—Li4—O41	101.46 (13)
O12—C17—C16	116.66 (11)	O14—Li4—O42	119.36 (13)
N11—C16—C15	123.40 (11)	O41—Li4—O42	113.99 (14)
N11—C16—C17	113.81 (10)	O14—Li4—O13^iv^	117.61 (13)
C15—C16—C17	122.76 (10)	O41—Li4—O13^iv^	98.62 (12)
C14—C15—C16	117.16 (11)	O42—Li4—O13^iv^	104.16 (13)
C14—C15—H15	121.4	O14—Li4—Li3^ii^	99.97 (11)
C16—C15—H15	121.4	O41—Li4—Li3^ii^	151.72 (13)
O23—C28—O24	127.23 (12)	O42—Li4—Li3^ii^	38.23 (8)
O23—C28—C23	116.20 (10)	O13^iv^—Li4—Li3^ii^	87.47 (11)
O24—C28—C23	116.54 (11)	O14—Li4—Li2^iv^	147.07 (12)
N22—C23—C24	122.97 (11)	O41—Li4—Li2^iv^	73.92 (10)
N22—C23—C28	114.46 (10)	O42—Li4—Li2^iv^	91.18 (10)
C24—C23—C28	122.55 (10)	O13^iv^—Li4—Li2^iv^	35.52 (7)
O14—C18—O13	126.36 (12)	Li3^ii^—Li4—Li2^iv^	97.25 (9)
O14—C18—C13	116.92 (11)	O14—Li4—H422	140.9 (7)
O13—C18—C13	116.71 (11)	O41—Li4—H422	101.3 (6)
N12—C13—C14	123.64 (11)	O42—Li4—H422	21.6 (6)
N12—C13—C18	114.30 (10)	O13^iv^—Li4—H422	89.7 (7)
C14—C13—C18	122.05 (11)	Li3^ii^—Li4—H422	50.9 (6)
C15—C14—C13	116.97 (11)	Li2^iv^—Li4—H422	70.4 (7)
C15—C14—H14	121.5	C25—C24—C23	117.20 (11)
C13—C14—H14	121.5	C25—C24—H24	121.4
O31—Li3—O22^iii^	103.19 (14)	C23—C24—H24	121.4
O31—Li3—O21	96.46 (12)	C24—C25—C26	117.37 (11)
O22^iii^—Li3—O21	125.74 (14)	C24—C25—H25	121.3
O31—Li3—O42^ii^	111.96 (14)	C26—C25—H25	121.3
O22^iii^—Li3—O42^ii^	107.33 (12)	O22—C27—O21	126.48 (12)
O21—Li3—O42^ii^	111.05 (13)	O22—C27—C26	117.17 (11)
O31—Li3—Li4^ii^	74.81 (11)	O21—C27—C26	116.35 (11)
O22^iii^—Li3—Li4^ii^	113.96 (11)	C27—O21—Li3	108.76 (11)
O21—Li3—Li4^ii^	119.92 (12)	C27—O21—Li1	117.39 (11)
O42^ii^—Li3—Li4^ii^	37.31 (7)	Li3—O21—Li1	133.16 (11)
O31—Li3—Li3^iii^	149.11 (18)	C27—O22—Li3^iii^	134.00 (13)
O22^iii^—Li3—Li3^iii^	57.89 (8)	Li3—O31—H311	122.0 (18)
O21—Li3—Li3^iii^	79.98 (11)	Li3—O31—H312	127 (2)
O42^ii^—Li3—Li3^iii^	97.71 (12)	H311—O31—H312	110 (3)

Symmetry codes: (i) −*x*+2, −*y*, −*z*+2; (ii) −*x*+2, −*y*+1, −*z*+2; (iii) −*x*+2, −*y*, −*z*+3; (iv) −*x*+2, −*y*+1, −*z*+1.

### Hydrogen-bond geometry (Å, °)

**Table:**

*D*—H···*A*	*D*—H	H···*A*	*D*···*A*	*D*—H···*A*
O31—H312···O14^v^	0.75 (3)	2.11 (3)	2.8572 (17)	171 (3)
O42—H421···O1	0.82 (3)	1.97 (3)	2.768 (2)	165 (3)
O42—H422···O23^iv^	0.86 (3)	1.95 (3)	2.7676 (16)	158 (2)
O1—H11···O24^vi^	0.91 (3)	2.00 (3)	2.9036 (19)	175 (2)
O31—H311···O11	0.81 (3)	1.92 (3)	2.7117 (17)	163 (2)
O41—H442···O14^vii^	0.84 (2)	2.10 (2)	2.9306 (18)	167 (2)
O41—H441···O12^viii^	0.84 (3)	1.92 (3)	2.7607 (16)	172 (2)

Symmetry codes: (v) *x*, *y*, *z*+1; (iv) −*x*+2, −*y*+1, −*z*+1; (vi) *x*−1, *y*+1, *z*; (vii) −*x*+1, −*y*+1, −*z*+1; (viii) *x*, *y*, *z*−1.
